# Outcomes among Mothers Who Gave Birth in the Health Facility: Does Birth Preparedness and Complication Readiness Have a Role?

**DOI:** 10.1155/2019/5147853

**Published:** 2019-04-30

**Authors:** Temesgen Worku Gudayu, Bilen Mekonnen Araya

**Affiliations:** University of Gondar, College of Medicine and Health Sciences, School of Midwifery, Gondar, Ethiopia

## Abstract

**Background:**

Giving childbirth is a natural reward for human beings to replace themselves to exist in the world. Despite all the efforts made to improve maternal health, maternal morbidity and mortality continue during childbirth. Hence, this study aimed to determine the proportion of maternal birth outcomes and identify their predictors among mothers who gave birth in hospitals.

**Method:**

A hospital-based cross-sectional study was conducted from April 9 to June 7, 2016, among 384 postnatal mothers in Debre Tabor Hospital. Randomly selected mothers were interviewed by trained data collectors. Data were checked for completeness, entered using Epi Info version 7, and analyzed using STATA 14 software. A multivariate logistic regression model was used to control confounders and identify predictors of maternal birth outcomes. Statistical significance was declared by adjusted odds ratio with a 95% confidence interval and a *p* value ≤0.05.

**Result:**

About 77% (95% CI: 72.9, 81.3) of the mothers had good maternal birth outcomes. Antenatal care utilization (AOR: 2.60; 95% CI: 1.16, 5.83); BPCR practice (AOR: 2.10; 95% CI: 1.12, 3.96); self-preference of health institution (AOR: 2.34; 95% CI: 1.11, 4.50); and mode of delivery: caesarean (AOR: 0.46; 95% CI: 0.23, 0.87), assisted breech (AOR: 0.17; 95% CI: 0.04, 0.69), and instrumental (AOR: 0.27; 95% CI: 0.09, 0.79) were associated with good maternal birth outcome.

**Conclusion:**

In this study, the maternal birth outcome among postnatal mothers was good in more than three-fourth of the cases. Hence, encouraging mothers to utilize health-care services and counseling and supporting them on BPCR practice are recommended.

## 1. Introduction

In the year 2015, an estimated rate of 303,000 maternal mortality had occurred globally [1]; maternal mortality ratio (MMR) has shown a global reduction from 385 (deaths/100,000 live births) in 1990 to 216 in 2015 [2].

Developing countries, as reported in 2015, are suffering from the high burden of maternal mortality with about 99% of global estimates [1], of which the largest proportion (about 66%) was noted in sub-Saharan Africa [2].

Maternal mortality trends in Ethiopia demonstrated a decreasing MMR from 871 in 2000 to 412 in 2016 [3]. The lifetime risk of maternal death related to pregnancy or childbirth as presented in this report is 21 in 1000 women in Ethiopia.

World strived to cope with maternal health problems and developed different strategies. The World Health Organization (WHO) developed a manual and suggested recommendations for maternal and neonatal health [4, 5], particularly on prenatal care and service integration. In addition, postnatal care for mothers and newborns recommended by the WHO is another dimension of care for the improvement of maternal and neonatal health [6].

Presence of a skilled attendant at birth is as well among key strategies to improve maternal and neonatal health [7]. The level of care obtained during the antenatal period [8, 9], in general, and birth preparedness and complication readiness (BPCR) intervention in return, in particular, increases the use of skilled birth attendants [10–12] and is believed to reduce maternal morbidity and mortality.

A systematic review and meta-analysis result revealed that disclosure to BPCR interventions resulted in a statistically significant fall of neonatal mortality risk by 18% and a nonsignificant reduction of maternal mortality risk by 28% [13]. A subgroup analysis in this review further identified that about 30% of women who participated in interventions showed a 24% significant reduction in neonatal mortality risk and a 53% significant reduction in maternal mortality risk.

Although all possible efforts to improve maternal health care are in place, maternal morbidity and mortality remain a challenge. Hence, this study was intended to determine the proportion and determinants of maternal birth outcomes among postnatal mothers who gave birth in Debre Tabor Hospital.

## 2. Methods

This hospital-based cross-sectional study was conducted among mothers who gave birth in Debre Tabor Hospital and admitted to the postnatal unit.

Debre Tabor is the capital of South Gondar administration zone and is 98 km northwest to Bahir Dar city. The hospital is situated in the middle of the zone and serves for an estimated 2,445,826 population of the zone. Besides all other services, the hospital provides an estimated rate of 500 delivery services per month.

The data were collected for a period of 2 months from April 9 to June 7, 2016. Mothers were approached within 24 hours of delivery and before discharge when stayed longer. All mothers who were admitted to the postnatal unit were considered as study population, and those who might develop some iatrogenic complications were treated under exclusion criteria.

The sample size was calculated based on the following assumptions: Mothers who were expected to give birth in a period of two months were estimated to be 1000. Proportions of mothers who were supposed to have good birth outcome were assumed to be 50% since previous studies which reported similar findings were lacking. Taking 5% margin of error, 95% confidence level, and single population proportion formula, the maximum sample size was turned to 384.

Systematic random sampling technique was used to select study participants. A skip interval of 3 was calculated; among the first three postnatal admissions, the 1^st^ mother was randomly selected; and thereafter, every third client was interviewed until the estimated sample size was obtained in a period of two months.

A pretested structured questionnaire was developed in English and translated to Amharic then back to English to maintain its consistency. Training on the aim of the study, data collection tool, and procedure was given for the data collectors and supervisor. A pretest was then conducted in a nearby health center among 20 postnatal mothers, and a minor correction was made on the questionnaire before actual data collection.

Three midwives, BSc, conducted a face-to-face interview, observation, and chart review. One midwife, BSc, supervised the data collection process. Data collectors interviewed participants on their sociodemographic, reproductive and obstetric, and BPCR-related data, whereas birth outcome data were collected through direct patient observation and chart review.

The maternal birth outcome was measured as “good” if mothers were clinically assessed normal and discharged within the recommended time, whereas those who developed postpartum hemorrhage and postpartum sepsis and sustained injury to the perineum, cervix, and bladder were categorized as having “bad” outcome.

Birth preparedness and complication readiness was measured based on the following five areas: identifying health institution for labor and delivery, saving money for labor- and delivery-related reasons, identifying skilled health-care provider(s) for labor and delivery, identifying means of transportation to place of childbirth or in case of emergency, and arranging blood donor in case if needed. Mothers who followed three and more steps of the above five components were considered prepared in this study which was also considered in previous studies [14–17].

Mothers who came to the current place of delivery by their own or their families' preference were considered as “self-referred/self-preference,” and those who were referred by the community or other lower-level health facilities were considered as “referred”.

The collected data were first manually checked for completeness and coded and then entered into Epi Info version 7 and then transported to STATA software version 14 for analysis. Descriptive analysis was done and presented using tables, figure, and text. Bivariate logistic regression analysis was done to identify the association of sociodemographic and reproductive and obstetric variables with the maternal birth outcome. Then, multivariate logistic regression was done by running all variables with a *p* value ≤0.2 under bivariate analysis in order to control the confounding variable and identify factors associated with the outcome variable. The strength of association was determined by the adjusted odds ratio (AOR) with its 95% confidence interval (CI) and a *p* value ≤0.05.

Written ethical clearance was obtained from the research and ethical committee of the Department of Midwifery, College of Medicine and Health Sciences, University of Gondar. Debre Tabor hospital was then communicated through a formal support letter and permission gained. Informed consent was obtained from participants, and they were assured about the privacy and confidentiality of their responses.

## 3. Result

### 3.1. Sociodemographic Characteristics of the Study Participants

Three hundred eighty-four mothers participated in the study, and their age ranged from 16 to 46 years (median age (interquartile) was 28 (8.5) years). The majority were married and Orthodox Christians. More than half of the participants were rural residents, and nearly three-fourth were housewives. About one-third of the mothers had not attended formal education, and one-fifth had education beyond secondary school (Table 1).

### 3.2. Obstetric Characteristics of Study Participants

About nine-tenths of the participants in this study had ANC during their index pregnancy, but only about one-third attended 4 visits. More than half of the mothers gave birth in the hospital because they were referred from other facilities. The majority gave vaginal birth, and spontaneous vaginal delivery occurred among three-fourth of the participants (Table 2).

### 3.3. Maternal Birth Outcome among Study Participants

**Figure 1:**
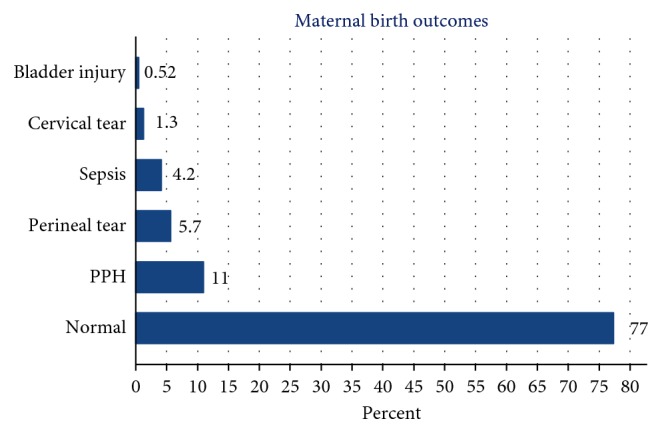
Percentage of maternal birth outcomes of postnatal mothers, Debre Tabor Hospital, 2016.

In about 77% of the mothers, the maternal birth outcome was normal, and they were discharged without having complications. The common complication (11%) in this study was postpartum hemorrhage (PPH), and about 4.2% developed postpartum sepsis. The remaining 7.5% sustained genital and urinary tract injuries (Figure 1).

### 3.4. Factors Associated with Maternal Birth Outcomes among Postnatal Mothers

In multivariate logistic regression analysis, attending ANC during index pregnancy is significantly associated with maternal birth outcome (AOR, 2.60; 95% CI: 1.16, 5.83). Well preparedness for birth and its complication and self-preference of health facility for labor and delivery were also associated with good maternal birth outcome. On the contrary, modes of delivery other than a spontaneous vaginal mode of delivery were protective with good maternal birth outcome (Table 3).

## 4. Discussion

This research has shown birth outcome proportions among postnatal mothers. In addition, it identified factors associated with good birth outcomes. Accordingly, nearly three-fourth of the mothers experienced good birth outcome. Antenatal care utilization, preparedness for birth and its complication, self-preference of health facility for labor and delivery, and mode of delivery were predictors of birth outcome in this study.

In this study, among hospital births, 77% of the mothers experienced normal birth outcome. The remaining nearly 23% of the mothers developed complications such as postpartum hemorrhage (PPH) (11%), postpartum sepsis (4.2%), and genital and urinary tract tear/injury (7.5%). During the study period and among studied mother, no maternal mortality was reported.

Postpartum hemorrhage (PPH) found to be more common in this study. Other studies reported the proportion as 24% in Italy [18], 12.5% in Brazil [19], 9% in Uganda [20], 4.2% in Nigeria [21], and 0.3% in the USA [22]. Large-scale and multicenter studies reported small proportion, whereas small-scale studies reported higher proportion, and the difference could be explained by sample size. Although the proportion differs from studies to studies, PPH remains a common postpartum complication.

Infection in general, postpartum infection (sepsis) in particular, is also a commonly reported type of cause of maternal morbidity. This study determined about 4% of cases of postpartum sepsis. Studies in Nigeria [23] and Uganda [24] also reported an equivalent 3% and 2% proportion of postpartum sepsis. Intrapartum and postpartum infection could be related to infection prevention practices, and 2 out of 9 facilities which fulfilled the minimum infection prevention requirement in Arba Minch district [25] could reflect the risk of sepsis in developing countries.

Antenatal care is a commonly provided service for pregnant mothers in Ethiopia [3]. This study identified that antenatal care utilization is associated with good birth outcomes. Mothers who attended antenatal care were more than two times likely to have good birth outcomes than those who had not attended. A Bolivian study [26] which identified an association of lack of antenatal care with maternal morbidity supports this finding. Antenatal care is designed to provide preventive interventions for mothers and is believed to prepare pregnant mothers for better outcomes. This concept is also supported by a meta-analysis [27] in which dietary and lifestyle interventions in pregnancy were associated with improved outcomes for both mothers and babies.

This study in addition identified that mothers who were well prepared for birth and complications were two times more likely to have good outcomes than those who were less prepared. Studies also supported that birth preparedness and complication readiness interventions besides contributing to maternal well-being were identified to contribute to reducing maternal and neonatal mortality in developing countries [13]. The association of self-preference of health facility with good birth outcome in this study further supports the contribution of BPCR with a good birth outcome.

The current study further identified that mode of delivery is associated with maternal birth outcomes. Compared to those who gave spontaneous vaginal delivery, mothers who delivered by caesarean section were 54% more likely to develop complications. Also, assisted breech and instrumental delivery, respectively, hold 83% and 73% more likely risk of complication than spontaneous vaginal delivery. Studies [28, 29] as well identified statistically significant likely risk of PPH and perineal tear among instrument-assisted vaginal deliveries compared with spontaneous vaginal delivery and PPH [30] among caesarean deliveries. This implies a delivery which failed to terminate naturally and needed further obstetrical assistance bears a higher risk of complication.

Although this study is limited to a single institution, the data quality is better since it was collected through interviewer-administered questionnaire and observation and the study tried to identify actionable factors to poor maternal birth outcomes. However, it would have been better if such studies became a multicenter and incorporated quality of delivery-care services.

## 5. Conclusion

In this study, maternal birth outcome among postnatal mothers was good in more than three-fourth of the cases. However, nearly a quarter of the mothers developed postpartum hemorrhage and postnatal sepsis and sustained genital tract injury. Antenatal care utilization, BPCR practice, self-preference of a hospital for labor and delivery service, and mode of delivery were statistically significant factors for good maternal birth outcomes. Hence, providing individual-, couple-, and community-level interventions in encouraging mothers to utilize health-care services and health facilities as well as counseling and supporting them on BPCR practice is recommended.

## Figures and Tables

**Table 1:** Frequency distribution of sociodemographic variables cross tabulated with maternal birth outcome, among postnatal mothers in Debre Tabor Hospital, 2016 (*n* = 384).

Variables	Maternal birth outcome		Total frequency (%)
	Good (%)	Poor (%)	
*Maternal age*			
<20 years	16 (4.2)	6 (1.6)	22 (5.8)
20–34 years	234 (60.9)	52 (13.6)	286 (74.5)
>34 years	47 (12.2)	29 (7.6)	76 (19.8)

*Marital status*			
In marital relation	279 (72.7)	78 (20.3)	357 (93.0)
Not in marital relation^*∗*^	18 (4.7)	9 (2.3)	27 (7.0)

*Religion*			
Orthodox	252 (65.6)	72 (18.8)	324 (84.3)
Muslim	38 (9.9)	13 (3.4)	51 (13.3)
Protestant	7 (1.8)	2 (0.5)	9 (2.3)

*Resident*			
Urban	147 (38.3)	26 (6.8)	173 (45.1)
Rural	150 (39.1)	61 (15.9)	211 (55.0)

*Educational status*			
Unable to read and write	95 (24.8)	44 (11.5)	139 (36.4)
Able to read and write	87 (22.6)	17 (4.4)	104 (27.0)
Primary education	44 (11.5)	16 (4.2)	60 (15.7)
Secondary and above	71 (18.5)	10 (2.6)	81 (21.1)

*Occupation*			
Housewife	208 (54.2)	63 (16.4)	271 (70.6)
Public employee	39 (10.2)	9 (2.3)	48 (12.)
Self-employed	50 (13.0)	15 (3.9)	65 (16.9)

*Family size*			
≤3	102 (26.6)	15 (3.9)	117 (30.5)
4–6	144 (37.5)	47 (12.2)	191 (49.7)
≥7	51 (13.3)	25 (6.5)	76 (19.8)

^*∗*^Single, widowed, divorced, or cohabited.

**Table 2:** Frequency distribution of obstetric variables cross-tabulated with maternal birth outcome, among postnatal mothers in Debre Tabor Hospital, 2016 (*n* = 384).

Variables	Maternal birth outcome		Total frequency (%)
	Good (%^*∗*^)	Poor (%^*∗*^)	
*Parity*			
Para 1	104 (88.1)	14 (11.9)	118 (30.7)
Para 2–4	137 (74.1)	48 (25.9)	185 (48.2)
Para ≥5	56 (69.1)	25 (30.9)	81 (21.1)

*Had ANC in the current pregnancy*			
Yes	277 (80.9)	65 (19.1)	342 (89.1)
No	20 (47.6)	22 (52.4)	42 (10.9)

*Number of ANC visits*			
0	20 (47.6)	22 (52.4)	42 (10.9)
1	15 (71.4)	6 (28.6)	21 (5.5)
2	66 (75.0)	22 (25.0)	88 (22.9)
3	81 (86.2)	13 (13.8)	94 (24.5)
4	115 (82.7)	24 (17.3)	139 (36.2)

*Preference of this hospital*			
Self-preference	144 (86.8)	22 (13.2)	166 (43.2)
Referred from another facility	153 (70.2)	65 (29.8)	218 (56.8)

*Mode of delivery*			
Spontaneous vaginal	230 (81.8)	51 (18.2)	281 (73.2)
Caesarean	45 (67.2)	22 (32.8)	67 (17.5)
Instrumental	14 (60.9)	9 (39.1)	23 (5.9)
Breech	8 (61.5)	5 (38.5)	13 (3.4)

^*∗*^Within row relative frequency percentage.

**Table 3:** Sociodemographic and obstetric factors associated with maternal birth outcome, among postnatal mothers in Debre Tabor Hospital, 2016 (*n* = 384).

Variables	Maternal birth outcome		COR (95% CI) *p* value	AOR (95% CI)
	Good (%)	Bad (%)		
*Residence*				
Urban	147 (38.3)	26 (6.7)	2.29 (1.38, 3.84)	1.15 (0.55, 2.41)
Rural	150 (39.1)	61 (15.9)	1	1

*Maternal education level*				
Unable to read and write	95 (24.7)	44 (11.5)	1	1
Able to read and write	87 (22.7)	17 (4.4)	2.37 (1.26, 4.45)	1.10 (0.51, 2.37)
Primary level (1–8) class	115 (29.9)	26 (6.7)	2.05 (1.18, 3.57)	0.45 (0.19, 1.07)

*Family size*				
≤3	102 (26.6)	15 (3.9)	3.33 (1.62, 6.87)	0.88 (0.19, 3.95)
4–6	144 (37.5)	47 (12.2)	1.50 (0.84, 2.68)	1.13 (0.42, 3.06)
≥7	51 (13.3)	25 (6.5)	1	1

*Had ANC*				
Yes	277 (72.1)	65 (16.9)	4.69 (2.42, 9.09)	**2.60 (1.16, 5.83)**
No	20 (5.2)	22 (5.7)	1	1

*The pregnancy was planned*				
Yes	255 (66.4)	56 (14.9)	3.36 (1.95, 5.81)	1.47 (0.74, 2.94)
No	42 (10.9)	31 (8.1)	1	1

*BPCR practice*				
Well practiced	188 (48.9)	31 (8.1)	3.12 (1.89, 5.13)	**2.10 (1.12, 3.96)**
Less practiced	109 (28.4)	56 (14.6)	1	1

*Preference of this hospital*				
Self-preference	144 (37.5)	22 (5.7)	2.78 (1.63, 4.74)	**2.34 (1.11, 4.50)**
Referred from another facility	153 (39.8)	65 (16.9)	1	1

*Parity*				
One	104 (27.1)	14 (3.7)	3.32 (1.59, 6.88)	4.71 (0.97, 22.93)
2–4	137 (35.7)	48 (12.5)	1.27 (0.72, 2.26)	0.86 (0.32, 2.34)
≥5	56 (14.6)	25 (6.5)	1	1

*Mode of delivery*				
Spontaneous vaginal	230 (59.9)	51 (13.3)	1	1
Caesarean	45 (11.7)	22 (5.7)	0.45 (0.25, 0.82)	**0.46 (0.23, 0.87)**
Assisted breech	8 (2.1)	5 (1.3)	0.35 (0.11, 1.13)	**0.17 (0.04, 0.69)**
Instrumental	14 (3.7)	9 (2.3)	0.34 (0.14, 0.84)	**0.27 (0.09, 0.79)**

ANC = antenatal care. BPCR = birth preparedness and complication readiness.

## Data Availability

The data used to support the findings of this study are available from the corresponding author upon request.
